# Tip dating and Bayes factors provide insight into the divergences of crown bird clades across the end-Cretaceous mass extinction

**DOI:** 10.1098/rspb.2023.2618

**Published:** 2024-02-14

**Authors:** Neil Brocklehurst, Daniel J. Field

**Affiliations:** ^1^ Department of Earth Sciences, University of Cambridge, Cambridge, UK; ^2^ Museum of Zoology, University of Cambridge, Cambridge, UK

**Keywords:** bird, end-Cretaceous extinction, tip-dating, phylogeny, Bayes factor

## Abstract

The origin of crown birds (Neornithes) remains contentious owing to conflicting divergence time hypotheses obtained from alternative sources of data. The fossil record suggests limited diversification of Neornithes in the Late Mesozoic and a substantial radiation in the aftermath of the Cretaceous–Palaeogene (K–Pg) mass extinction, approximately 66 Ma. Molecular clock studies, however, have yielded estimates for neornithine origins ranging from the Early Cretaceous (130 Ma) to less than 10 Myr before the K–Pg. We use Bayes factors to compare the fit of node ages from different molecular clock studies to an independent morphological dataset. Our results allow us to reject scenarios of crown bird origins deep in the Early Cretaceous, as well as an origin of crown birds within the last 10 Myr of the Cretaceous. The scenario best supported by our analyses is one where Neornithes originated between the Early and Late Cretaceous (*ca* 100 Ma), while numerous divergences within major neoavian clades either span or postdate the K–Pg. This study affirms the importance of the K–Pg on the diversification of modern birds, and the potential of combined-evidence tip-dating analyses to illuminate recalcitrant ‘rocks versus clocks’ debates.

## 1. Introduction

**Figure 1.**
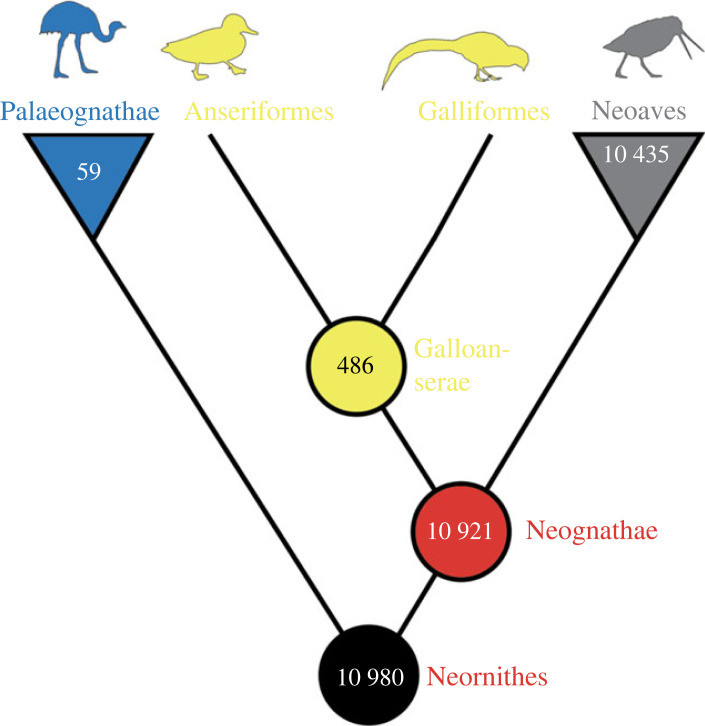
Schematic phylogeny illustrating interrelationships of the major avian clades under investigation; extant diversity following the International Ornithological Congress (IOC) World Bird List 13.2 [1] illustrated at nodes. Silhouettes modified from [2].

Crown birds (Neornithes) represent one of the most diverse extant vertebrate clades, with more than 10 000 living species (figure 1). However, our understanding of the earliest stages of neornithine evolution is incomplete, with debate surrounding the timing of crown bird origins owing to a sparse early fossil record [3–5]. These debates obscure the nature and pace of the early crown bird radiation, as well as the influence of the Cretaceous–Palaeogene (K–Pg) mass extinction on patterns of avian survivorship and diversification.

Disagreements regarding the age of the avian crown group have often been cited as a classic example of a ‘rocks versus clocks’ debate: whereas a direct reading of the fossil record suggests a neornithine radiation in the wake of the end-Cretaceous mass extinction, molecular clock analyses often infer numerous neornithine divergences occurring before the K–Pg. In reality, the debate is more nuanced: molecular divergence time estimates for the origin of Neornithes range from more than 130 Ma [6–8], well into the Early Cretaceous, to less than 72 Ma [9], only six million years before the K–Pg transition. Meta-analysis of published molecular clock studies have identified numerous potential confounding factors impacting divergence time estimates, including issues of data type (e.g. the use of mitochondrial versus nuclear data in divergence time analyses) and ‘epoch effects’ induced by directional selection on body size and life-history factors (e.g. [10,11]).

Although a range of Mesozoic fossils have at times been assigned to Neornithes (e.g. [12–14]), these are almost all exceptionally fragmentary [5], and the characters used to assign these fossils to crown birds are often dubious [15]. The affinities of the Maastrichtian (latest Cretaceous) fossil *Vegavis iaai*, hypothesized by Clarke *et al*. [16] to represent a crown anseriform, have been the subject of frequent debate, with positions both within and outside the avian crown group supported by alternative phylogenetic analyses (e.g. [17–21]). The recent discovery of *Asteriornis maastrichtensis* [21] may provide a less contentious record of Neornithes as early as the Late Maastrichtian (66.7–66.8 Ma), although whether *Asteriornis* represents a crown or stem galloanseran remains unclear [21,22]. Regardless, a position for *Asteriornis* within the galloanseran total clade logically implies that at least four crown bird subclades must have diverged from each other no later than the K–Pg boundary: the total clades of Palaeognathae and Neognathae, as well as the total clades of Galloanserae and Neoaves within neognaths.

Lee *et al*. [23] attempted to reconcile avian molecular clock ages with the fossil record via a ‘morphological clock’: using the principles espoused in molecular divergence dating (simultaneously inferring tree topologies and divergence dates using molecular sequence data and models of molecular evolution within a Bayesian framework) but applied to discrete morphological character data. Employing a variety of root age constraints and models of character evolution, they found the majority of early crown bird divergences to have occurred during the mid-Late Cretaceous, considerably earlier than the first appearances of these lineages in the fossil record. Their estimate of the age of the neornithine root node lay between 99 and 119 Ma, consistent with many molecular clock estimates.

Morphological tip-dating approaches have become widely used tools for palaeontologists in recent years. The development of the fossilized birth–death (FBD) model [24,25] provides a method for analysing morphological datasets in an evolutionary framework, incorporating not only morphological data into phylogenetic inference, but estimates of sampling probabilities, extinction rates and speciation rates based on fossil occurrence data. Bayes factors enable comparisons of results of Bayesian analyses produced using different priors, to see which best fit the observed data. In the context of phylogenetic analysis, Bayes factors have been used to test the impact of alternative substitution models [26,27], models of rate variation [27–29], competing phylogenetic hypotheses [21,30] and uncertainty surrounding the ages of geological formations [31]. Here, for the first time to our knowledge, we use this useful probabilistic approach to compare age priors for nodes deep within the neornithine tree of life drawn from alternative molecular divergence time studies. This approach is intended to help clarify the antiquity of the avian crown group, and the influence of the K–Pg mass extinction event on neornithine diversification patterns—two of the most contentious outstanding research topics in avian macroevolution.

## 2. Material and methods

### (a) Dataset

We used the character/taxon dataset from Field *et al*. [21], as it contains both fossil and extant representatives of all major neornithine clades: Palaeognathae, Galloanserae and Neoaves. Its fossil sampling also includes the earliest known representatives of Neornithes, providing the best possible constraints on divergences across the K–Pg boundary.

Neornithes and the non-crown avialan lineages prevalent during the Late Cretaceous have generally been subjected to phylogenetic analysis in isolation of each other. To reliably constrain the neornithine root age, a comprehensive sample of lineages surrounding the neornithine root node is necessary, including K–Pg representatives of Neornithes as well as crownward stem bird outgroups to constrain the timing of the divergences predating the neornithine root. To resolve this issue, Mesozoic outgroup taxa were added to the matrix for the FBD analysis. The stem bird taxa investigated, as well as their interrelationships and estimated divergence times, were taken from the analysis of Brocklehurst & Field [32].

### (b) Fossilized birth–death analysis

The resultant matrix of stem and crown birds was subjected to Bayesian tip dating analysis using the FBD model implemented in MrBayes 3.2.6 [33]. To account for temporal uncertainty in the first appearances of fossil taxa, taxon ages were represented by a uniform probability distribution covering the full uncertainty of the age of the geological formation or assemblage zone in which they first appear. An independent gamma rates model was employed to account for rate heterogeneity among branches. Rate heterogeneity among characters was also modelled as a gamma distribution. The analysis was carried out with two runs containing two chains for 50 million generations, sampling every 1000, with 25% of samples discarded as burn-in.

Node age priors were assigned to the crown groups of Neoaves, Galloanserae, Neognathae, Palaeognathae and Neornithes. Truncated normal distributions were employed, with the mean and standard deviations of ages taken from molecular clock studies and the minimum ages representing the ages of the oldest known fossils belonging to each crown clade. Four sets of node age priors were used, based on alternative molecular divergence time studies positing markedly different temporal scenarios for early crown bird evolution: Brown *et al*. [7], where the majority of early neornithine divergences occur in the Early Cretaceous (hereafter referred to as the Brown priors); Jarvis *et al*. [34], where early neornithine divergences occurred in the Late Cretaceous with diversification in the Early Palaeogene (hereafter the Jarvis priors); Prum *et al*. [9], where the initial neornithine divergences occurred in the latest Cretaceous, and the majority of divergences occurred in the Palaeogene (hereafter the Prum priors); and a set of priors where divergence times for the major neornithine subclade Neoaves were drawn from Prum *et al*. [9] and all others from Jarvis *et al*. [34] (hereafter the J&P priors).

As employed by Field *et al*. [21], partial topological constraints were assigned to ensure that the phylogenetic inter-relationships of extant taxa were consistent with topologies obtained from molecular data, but the position of fossil taxa could be inferred based on the analysis of morphological data. However, a modification was required as MrBayes does not allow nodes with partial topology constraints to be assigned node age priors. Therefore, full constraints were employed on the nodes for which age priors were assigned (Neoaves, Galloanserae, Neognathae, Palaeognathae and Neornithes), with fossil taxa assigned to the clades in which they were found by Field *et al*. [21]. Since the precise affinities of *Asteriornis* remain unclear [21], two sets of analyses were carried out: one where *Asteriornis* was constrained within crown Galloanserae, and one where it was constrained within crown Neognathae, but outside crown Galloanserae.

### (c) Bayes factor calculation

The results of the analyses were compared via Bayes factors. Stepping-stone analyses were used to infer the marginal likelihood of each hypothesis in MrBayes; these refer to a series of Markov chain Monte Carlo simulations that iteratively sample from probability distributions forming discrete steps between the posterior and prior distributions, placing increasing emphasis on priors, to see if hypotheses more consistent with the prior assumptions produce trees with higher likelihoods [35]. The Bayes factor is calculated as double the difference in log likelihoods between two hypotheses.

## 3. Results and discussion

The Bayes factors comparisons produce reasonably consistent results depending on the position of *Asteriornis* within or outside the galloanseran crown group. The Brown priors receive extremely poor support when compared with others, and the Jarvis priors are consistently better supported than the Prum priors (table 1). Using the criteria defined by Kass & Raftery [36], support for the Jarvis priors over both the Prum and the Brown priors is ‘very strong’. Under all four sets of priors tested, the analyses where *Asteriornis* was constrained to a position within the galloanseran crown received the highest likelihood, consistent with the original analyses of Field *et al.* [21].

**Table 1.** Likelihoods and Bayes factor comparisons of the tip-dating analyses with four sets of age priors and two sets of topology constraints.

priors		log likelihood	Bayes factors		
			Brown	Jarvis	Prum
*Asteriornis* within crown Galloanserae	Brown	−9254.88
	Jarvis	−9240.65	28.46
	Prum	−9247.18	15.4	13.06
	J&P	−9240.21	29.34	0.88	13.9
*Asteriornis* outside crown Galloanserae	Brown	−9257.69
	Jarvis	−9241.09	33.2
	Prum	−9247.73	19.92	13.28
	J&P	−9248.66	18.06	15.14	1.86

The two sets of analyses, varying in the position of *Asteriornis*, yield differing results regarding the J&P priors. When *Asteriornis* is placed within crown Galloanserae, the J&P priors receive slightly greater support than the Jarvis priors (table 1). The difference in support is negligible; according to the criteria of Kass & Raftery [36], it is ‘not worth more than a bare mention’. However, where *Asteriornis* is constrained to be outside crown Galloanserae, the J&P priors receive substantially less support than the Jarvis priors, and less also than the Prum priors. For all sets of priors, the best-supported position of *Asteriornis* is within the galloanseran crown, as a stem galliform.

**Figure 2.**
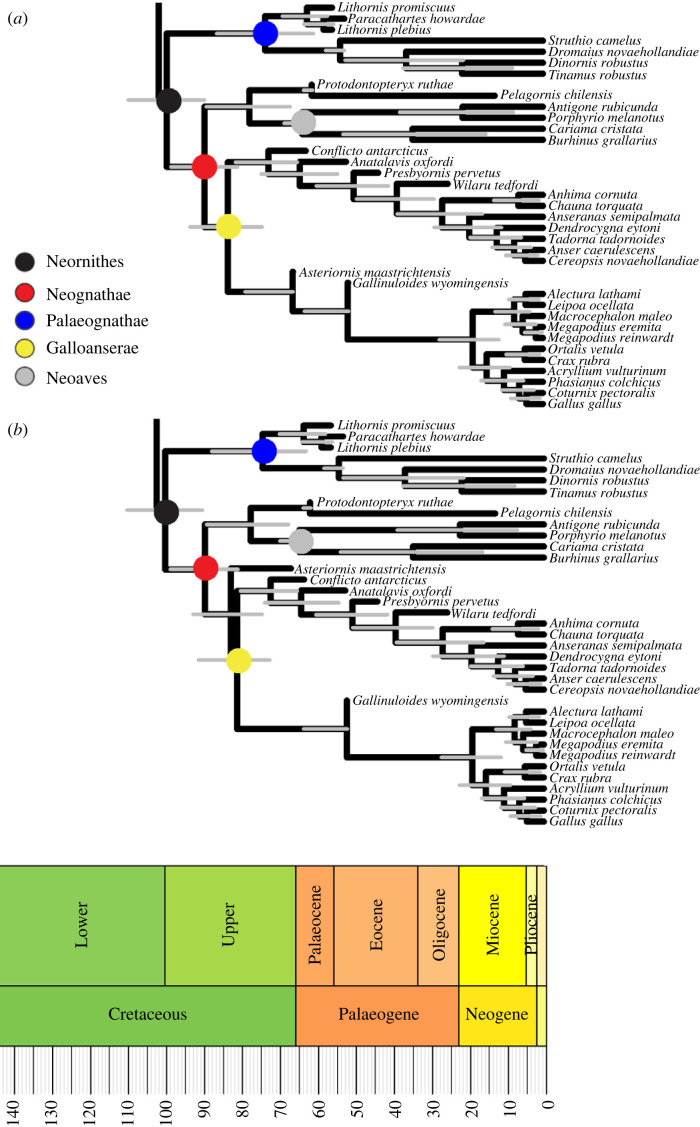
Maximum clade credibility trees produced by tip dating analysis using the fossilized birth–death model, with the best fitting set of node age priors according to Bayes factors. Dots at nodes indicate the major clades. (*a*) *Asteriornis* is constrained to a position within crown Galloanserae, J&P node age priors; (*b*) *Asteriornis* is constrained to a position within crown Neognathae but outside crown Galloanserae, Jarvis node age priors.

**Figure 3.**
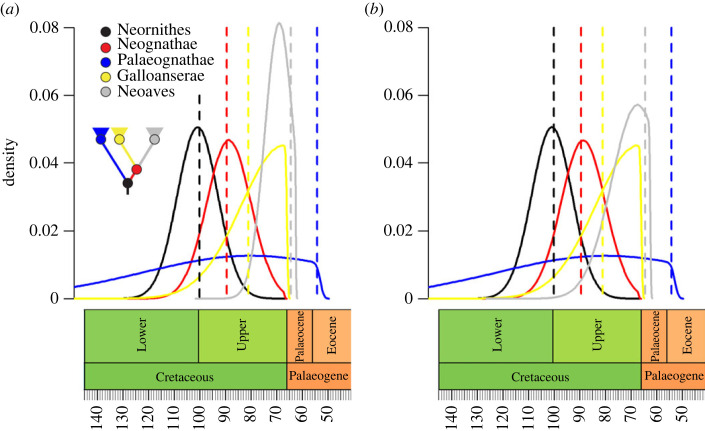
Illustrations of the node age prior probabilities found to fit the observed data best, and median inferred ages of the principal neornithine clades. Density distributions represent the shape of the node-age priors used in the fossilized birth–death analysis; dashed lines represent the median inferred age. (*a*) *Asteriornis* is constrained to a position within crown Galloanserae, J&P node age priors; (*b*) *Asteriornis* is constrained to a position within crown Neognathae but outside crown Galloanserae, Jarvis node age priors.

Nevertheless, the maximum clade credibility tree produced with the best-supported sets of priors for each position of *Asteriornis* yields little difference in the estimated age of the major neornithine subclade Neoaves (figure 2). Although the Prum *et al*. [9] study did find a younger age than Jarvis *et al*. [34] for the origin of crown Neoaves, they also estimated wider confidence intervals around this age estimate, so the age priors overlap (figure 3). Under these circumstances, both the Jarvis and the J&P priors support a divergence age of the neoavian crown of just over 64 Ma, with a confidence interval straddling the K–Pg (figure 2). When no character data are used and node ages are inferred entirely by sampling and the birth and death parameters, the divergence ages inferred for Neornithes and Neognathae are younger than those inferred with character data (electronic supplementary material, figures S1 and S2), but still well within the middle Late Cretaceous, older than inferred by Prum *et al*. [9].

The phylogenetic positions of other fossil taxa were generally aligned with the results of recent studies. Notably, the phylogenetically problematic extinct clade Pelagornithidae was not found to be a total-group galloanseran as in previous analyses [37,38], but was found in both analyses to represent a lineage of stem neoavians (figure 2), as found by Field *et al*. [21] using the same dataset but different optimization criteria and priors.

The results obtained here support a scenario of neornithine diversification where crown Neornithes, Neognathae and Galloanserae all originate in the Late Cretaceous, with the age of the avian crown group estimated at between 90.3 and 110.5 Ma. Interestingly, despite using priors based on the dates obtained by Jarvis *et al*. [34], the median ages obtained for the origin of crown Palaeognathae and Neoaves are younger than suggested by the original molecular divergence time study, although still within its reported margin of error (figure 3). Jarvis *et al*. [34] suggested that both of these crown clades originated during the latter stages of the Cretaceous, but our FBD analysis suggests divergence time estimates with margins of error straddling the K–Pg. Such discrepancies are not inconceivable; the only hard limit placed on node ages via the priors are minimum ages, and given sufficient data a Bayesian analysis can push results away from the mean value of a prior. The fact that the FBD model yielded younger ages for these two clades than the molecular age estimates specified in the priors could mean that rates of morphological change were distinct from those of molecular change at that point of avian evolutionary history, as suggested for eutherian mammals during the same general time interval [39], potentially underscoring similarities in the diversification histories of neoavian birds and eutherians with respect to the K–Pg [40,41].

Our results reject the two extreme sets of divergence dates for crown bird origins. The priors taken from Brown *et al*. [7], which suggest that many higher-order neornithine divergences occurred throughout the Cretaceous—with initial divergences occurring as early as the Valanginian (133 ± 8.1 Ma)—perform poorly under our assessment (table 1). To account for the lack of fossil evidence in line with this hypothesis, Brown *et al*. [7] posited a substantial radiation of neornithines in the Southern Hemisphere, which is comparatively undersampled from a palaeontological perspective, and that this early radiation may not have involved substantial morphological change such that early neornithines may not be distinguishable from Mesozoic stem bird lineages. A similar explanation had been posited for the ‘rocks versus clocks’ discrepancy regarding eutherian mammal origins [39,42]; however, our results reject such deep Mesozoic divergences for crown birds.

Although the Brown priors are rejected, the support for the Jarvis priors does still imply more than 25 Myr of unsampled neornithine history, raising concerns about the completeness of the fossil record of Mesozoic birds and its adequacy for macroevolutionary analyses. The avian fossil record consists of large numbers of fragmentary specimens and is dominated by a few areas of exceptional preservation [5,10]. The fossil record is particularly patchy across the K–Pg boundary, with only North American localities providing substantial sampling [43]. Phylogenetic comparative methods allow incomplete sampling to be acknowledged, and the FBD model explicitly incorporates incomplete sampling into its calculations. However, accounting for specific biases such as lagerstätte effects and geographical sampling requires the incorporation of assumptions from a user, and although feasible, can lead to overly complex, over-parametrized models [44]. The analyses included here therefore did not incorporate any attempt to account for specific variations in rates of sampling, extinction or origination through time. It is not certain how this might affect the results but it is likely that the analyses will be more conservative about inferring rapid divergences close to the end-Cretaceous extinction event (where high extinction rates and a subsequent radiation would lead to divergences being concentrated if rates are allowed to vary), and more likely to push divergences deeper. This makes the rejection of the Brown priors even more convincing.

In addition to the poor performance of the early-diverging scenario supported by the Brown priors, we also reject the much shallower divergences found by Prum *et al*. [9], where neornithine origins occurred only in the lattermost stages of the Cretaceous, less than 10 Myr before the K–Pg (table 1). Although our results support a scenario in which the majority of higher-order neornithine diversification occurred in the aftermath of the end-Cretaceous mass extinction event, congruent with the Prum priors, our results support an origin of Neornithes deeper in the Cretaceous, implying that the as-yet unsampled stem lineages of Neognathae and Palaeognathae persisted throughout the Late Cretaceous alongside a wide diversity of non-crown avialan lineages (e.g. [2,43,45,46]).

## 4. Conclusion

Our understanding of the origin of crown birds has been clouded by apparent conflicts between an observed Early Cenozoic radiation in the fossil record and markedly older ages obtained from molecular divergence time approaches. Tip dating approaches incorporating morphological data provide a means of reconciling these two sources of data, allowing both extant and extinct taxa to be analysed under an evolutionary model incorporating incomplete sampling in the fossil record. The FBD model also provides the means for explicitly testing alternative diversification scenarios via Bayes factors.

Our analysis of morphological data including both extant and extinct taxa (including a broad sample of neornithine and non-crown avialan lineages) rejects an origin of crown birds occurring deep in the Early Cretaceous. It also rejects the scenario at the opposite extreme: an origin of crown birds occurring less than 10 Myr before the end of the Cretaceous Period. Instead, the pattern of diversification best supported by the morphological data is one where neornithines originate between the Early and Late Cretaceous. Crown Galloanserae originates during the Late Cretaceous, while crown Palaeognathae and crown Neoaves diverge across the K–Pg boundary (figure 1).

Only the fossil record can provide direct evidence of the earliest stages of crown bird evolutionary history. However, considering the ongoing scarcity of this direct evidence, improved inferences based on models of molecular and morphological evolution are necessary to shed light on the Mesozoic origins of crown birds. Our results corroborate the hypothesized importance of the K–Pg in driving the extant bird radiation [9–11,34,40,47–51], and demonstrate the potential for Bayesian tip dating approaches for evaluating discrepancies between inferences based on morphological and molecular data.

## Data Availability

The data are provided in the electronic supplementary material [52].
